# Zn^II^ and Cu^II^-Based Coordination Polymers and Metal Organic Frameworks by the of Use of 2-Pyridyl Oximes and 1,3,5-Benzenetricarboxylic Acid

**DOI:** 10.3390/molecules26020491

**Published:** 2021-01-18

**Authors:** Ioannis Mylonas-Margaritis, Julia Mayans, Patrick McArdle, Constantina Papatriantafyllopoulou

**Affiliations:** 1 School of Chemistry, College of Science and Engineering, National University of Ireland Galway, University Road, H91 TK33 Galway, Ireland; i.mylonas-margaritis1@nuigalway.ie (I.M.-M.); patrick.mcardle@nuigalway.ie (P.M.); 2 Instituto de Ciencia Molecular (ICMol), Universidad de Valencia, Catedrático José Beltran 2, 46980 Paterna Valencia, Spain; julia.mayans@qi.ub.edu

**Keywords:** coordination polymers, metal-organic frameworks (MOFs), carboxylates, pyridyl oximes, zinc, copper

## Abstract

The simultaneous use of 2-pyridyl oximes (pyridine-2 amidoxime, H_2_pyaox; 2-methyl pyridyl ketoxime, Hmpko) and 1,3,5-benzenetricarboxylic acid (H_3_btc) provided access to five new compounds, namely [Zn(H_2_btc)_2_(H_2_pyaox)_2_]•2H_2_O (**1**•2H_2_O), [Zn(Hbtc)(H_2_pyaox)_2_]_n_ (**2**), [Cu(Hbtc)(H_2_pyaox)]_n_ (**3**), [Cu(Hbtc)(HmpKo)]_n_ (**4**) and [Cu_2_(Hbtc)_2_(Hmpko)_2_(H_2_O)_2_]•4H_2_O (**5**•4H_2_O). Among them, **3** is the first example of a metal-organic framework (MOF) containing H_2_pyaox. Its framework can be described as a 3-c uninodal net of **hcb** topology with the layers being parallel to the (1,0,1) plane. Furthermore, **3** is the third reported MOF based on a 2-pyridyl oxime in general. **2** and **4** are new members of a small family of coordination polymers containing an oximic ligand. **1–5** form 3D networks through strong intermolecular interactions. Dc magnetic susceptibility studies were carried out in a crystalline sample of **3** and revealed the presence of weak exchange interactions between the metal centres; the experimental data were fitted to a theoretical model with the fitting parameters being *J* = −0.16(1) cm^−1^ and *g* = 2.085(1). The isotropic *g* value was also confirmed by electronic paramagnetic resonance (EPR) spectroscopy. Reactivity studies were performed for **3** in the presence of metal ions; the reaction progress was studied and discussed for Fe(NO_3_)_3_ by the use of several characterization techniques, including single crystal X-ray crystallography and IR spectroscopy.

## 1. Introduction

The synthesis and characterization of metal coordination polymers and metal-organic frameworks (MOFs) have attracted a considerable research interest worldwide over the last decades, which stems from their technological, environmental and biomedical applications in sensing, catalysis, imaging, drug delivery, etc. [1,2,3,4,5,6,7,8,9,10]. Such species consist of mononuclear or low nuclearity inorganic units, which are linked through organic linkers with the nature of the metal ions and the organic linkers affecting their structure, having also the potential to introduce additional functionalities and physical properties in to the framework, e.g., photoluminescence, magnetism. For example, some 1D coordination polymers exhibit single chain magnetism (SCM) behaviour, and are promising candidates for applications in quantum computing, high-density information storage, etc. [11,12,13,14,15,16,17,18,19,20,21,22,23,24]. As the dimensionality of the framework increases, the induced porosity is combined in a synergistic way with its other physical properties, leading to the formation of hybrid multifunctional materials, with enhanced performance in a variety of applications (spintronics, photonics, catalysis and others).

Restricting further discussion to MOFs [8,9,10], they display a wide range of desirable structural features, such as large surface area, high porosity, flexible structure, stability and the possibility of controlled and targeted introduction of functional groups into the framework. Furthermore, some MOFs possess phenomena that affect their porosity and stability, namely the interpenetration and the breathing effect [25,26,27,28,29]. The former refers to the cases where more than one networks are catenated with each other [25,26,27], which potentially results in the increase of the surface area and stability of the MOF, and in the decrease of its pore diameter. The breathing effect is related to the change of the MOF pore dimensions upon encapsulation of a guest molecule as a result of the change of the intermolecular interactions [28,29]. The unique properties of MOFs and their structural tunability make these materials especially suitable for encapsulating a plethora of different guest molecules.

The wide range of applications of MOFs and coordination polymers constitute an increasing need for the development of efficient synthetic approaches towards new species with enhanced porosity and stability. The vast majority of MOFs has been synthesized using solvothermal techniques, while other approaches have been developed in recent years, based on post-synthetic, isoreticular, microwave-assisted, mechanochemical and sonochemical synthesis [30,31,32,33,34,35,36,37,38]. Each of these synthetic approaches often leads to different species, whose properties are merely dictated by the organic ligands that link the neighbouring SBUs, and by the nuclearity and type of the metal ion that is present in the structure. The organic linkers possess suitable coordination sites for the formation of the framework, providing the desirable flexibility and stability. The hydrogen bonding, π–π stacking, and other intermolecular interactions also affect the architecture of the overall framework and the MOF selectivity towards specific guest molecules. A large number of organic linkers has now been employed in MOFs synthesis including imidazolates, pyridine, carboxylates, etc., with the latter being one of the most commonly used, resulting in MOFs with a wide range of pores sizes and shapes [39,40,41,42,43,44,45,46,47,48,49]. The combination of two different linkers has been also employed for the synthesis of new MOFs; in this case, one of the ligands often plays the role of the pillar that link parallel layers leading to the formation of pillar-layered MOFs with increased dimensionality and novel topologies [50,51].

**Scheme 1:**
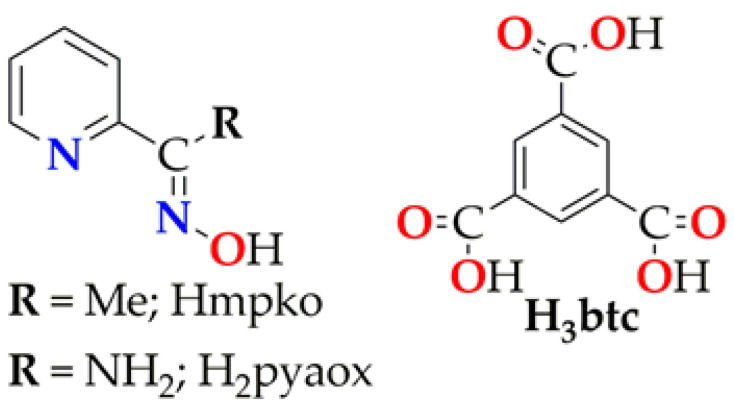
Schematic representation of the 2-pyridyl oximes (**left**) and the ligand H_3_btc (**right**) discussed in this work.

Although the impact of the ligand properties on the MOF topology and porosity has been well investigated, this is not the case for the nuclearity and properties of the SBU itself. To this end, we recently decided to introduce for the first time into the field of MOFs the 2-pyridyl oximes (Scheme 1), a family of ligands with high bridging capability, that have the potential to lead to high nuclearity species with unprecedented metal topologies. 2-pyridyl oximes have been extensively investigated in metal cluster chemistry and led to a plethora of metal clusters with interesting magnetic properties, including single-molecule and single-chain magnetism behaviour [52,53,54,55,56,57,58,59,60,61,62,63,64,65].

The initial combination of 2-pyridyl oximes with a variety of di-, tri- and tetra- carboxylic acids led to the first 2-pyridyl oxime- based MOFs, and other coordination polymers as well [66,67]. It is worth to mention that among the MOFs that we isolated with these ligands, [Cu_4_(OH)_2_(pma)(mpko)_2_]_n_, where pma^4-^ is the tetra-anion of 1,2,4,5-benzene tetracarboxylic acid (pyromellitic acid) and mpko- is the anionic form of 2-methyl pyridyl ketoxime, is based on a tetranuclear, butterfly-shaped SBU and possesses a novel 3,4,5,8-c net topology [66]. It also exhibits selectivity for Fe^3+^ adsorption with its magnetic properties being strongly related to the amount of metal ion present into the MOF pores [66].

The promising preliminary results of the employment of 2-pyridyl oximes in the field of MOFs prompted us to explore further this synthetic approach and we, herein, report the synthesis and characterization of five new compounds, by the use of a 2-pyridyl oxime (pyridine-2 amidoxime, H_2_pyaox and 2-methyl pyridyl ketoxime, Hmpko) in combination with 1,3,5-benzenetricarboxylic acid, H_3_btc (Scheme 1). Amongst the reported compounds, [Cu(Hbtc)(H_2_pyaox)]_n_ (3) is the first MOF bearing H_2_pyaox. [Zn(Hbtc)(H_2_pyaox)_2_]_n_ (2) and [Cu(Hbtc)(HmpKo)]_n_ (4) are rare examples of coordination polymers containing a 2-pyridyl oxime either in its neutral or anionic form. Note that H_3_btc has been employed in the field of MOFs and has led to the synthesis and characterization of many such species, including the MOFs HKUST, MIL-100, etc., [39,43,68,69,70,71,72,73,74,75,76,77], however its combination with an oximic ligand is essentially unexplored.

## 2. Results and Discussion

### 2.1. Synthetic Discussion

Our group has developed an intense interest over the last years in the synthesis of coordination polymers and MOFs by the use of 2-pyridyl oximes and a polycarboxylic ligand, such as 1,4-benzenedicarboxylic, 1,2,4,5-benzene tetracarboxylic acid, etc. [66,67]. These research efforts have resulted in new species, some of which display new framework topologies and promising sensing properties. It is worth to mention that the initial employment of 1,3,5-benzenetricarboxylic acid (H_3_btc) in Ni^II^ chemistry in the presence of an oximic ligand has led to the isolation of a 1D coordination polymer [67]. This prompted us to further study this reaction system by the use of different metal ions and investigate its potential to favour the formation of higher dimensionality coordination polymers and/or MOFs. A wide range of experiments was carried out in order to study the impact of the different synthetic parameters (presence/absence or kind of base, metal ratio of the reactants, metal sources, etc.) on the identity and crystallinity of the isolated product.

The reaction mixture of Zn(ClO_4_)_2_•6H_2_O/H_2_pyaox/H_3_btc (1:2:1) in H_2_O at 100 °C gave a colourless solution from which crystals of [Zn(H_2_btc)_2_(H_2_pyaox)_2_]•2H_2_O (**1**•2H_2_O) were subsequently isolated. Following a similar reaction but using a different Zn^II^ source (Zn(O_2_CMe)_2_•6H_2_O instead of Zn(ClO_4_)_2_•6H_2_O) and in the presence of NEt_3_, the 1D coordination polymer [Zn(Hbtc)(H_2_pyaox)_2_]_n_ (**2**) was isolated in good yield. The stoichiometric equation of the reactions that lead to the formation of **1** and **2** is represented in Equations (1) and (2). Note that the kind of base does not have any impact on the identity of the isolated compound, but it affects its crystallinity.

$$Zn{\left(ClO_{4}\right)}_{2}\cdot 6H_{2}O+2\;H_{2}pyaox+2\;H_{3}btc\;\overset{H_{2}O}{\to }\;$$

(1) $$\left[Zn{\left(H_{2}btc\right)}_{2}{\left(H_{2}pyaox\right)}_{2}\right]\cdot 2H_{2}O+4\;H_{2}O+2\;HClO_{4}$$

$$1\cdot 2H_{2}O\;$$

$$Zn{\left(O_{2}CMe\right)}_{2}\cdot 6H_{2}O+2\;H_{2}pyaox+H_{3}btc+2\;NEt_{3}\;\overset{H_{2}O}{\to }\;$$

(2) $${\left[Zn\left(Hbtc\right){\left(H_{2}pyaox\right)}_{2}\right]}_{n}+4\;H_{2}O+2HNEt_{3}{}^{+}+2\;MeCO_{2}{}^{-}$$

 2 

As a next step, we decided to investigate the impact of the metal ion on the identity of the isolated product; thus, by the use of Cu(ClO_4_)_2_•6H_2_O and following a similar synthetic approach to the one that provided access to **1**, green crystals of the 2D coordination polymer [Cu(Hbtc)(H_2_pyaox)]_n_ (**3**) were isolated in good yield. It is noteworthy that the electronic properties of the oximic ligand influence the dimensionality of the reaction product. In particular, by the employment of Hmpko instead of H_2_pyaox and by following the same reaction that yielded **3**, blue crystals of the 1D coordination polymer [Cu(Hbtc)(HmpKo)]_n_ (**4**) were formed, whereas the use of a different Cu^II^ source (Cu(NO_3_)_2_•2.5H_2_O instead of Cu(ClO_4_)_2_•6H_2_O) provided access to the compound [Cu_2_(Hbtc)_2_(Hmpko)_2_(H_2_O)_2_] 4H_2_O (**5**•4H_2_O), according to the stoichiometric Equations (3)–(5):

$$Cu{\left(ClO_{4}\right)}_{2}\cdot 6H_{2}O+\;H_{2}pyaox+\;H_{3}btc\;\overset{H_{2}O}{\to }\;$$

(3) $${\left[Cu\left(Hbtc\right)(H_{2}pyaox)\right]}_{n}+6\;H_{2}O+2\;HClO_{4}$$

3 

$$Cu{\left(ClO_{4}\right)}_{2}\cdot 6H_{2}O+\;Hmpko+\;H_{3}btc\;\overset{H_{2}O}{\to }\;$$

(4) $${\left[Cu\left(Hbtc\right)\left(Hmpko\right)\right]}_{n}+6\;H_{2}O+2\;HClO_{4}$$

4 

$$2\;Cu{\left(NO_{3}\right)}_{2}\cdot 2.5H_{2}O+\;2Hmpko+\;2H_{3}btc+H_{2}O\;\overset{H_{2}O}{\to }\;$$

(5) $$\left[Cu_{2}{\left(Hbtc\right)}_{2}{\left(Hmpko\right)}_{2}{\left(H_{2}O\right)}_{2}\right]\cdot 4H_{2}O+4\;HNO_{3}$$

$$5\cdot 4H_{2}O$$

### 2.2. Description of Structures

**Figure 1:**
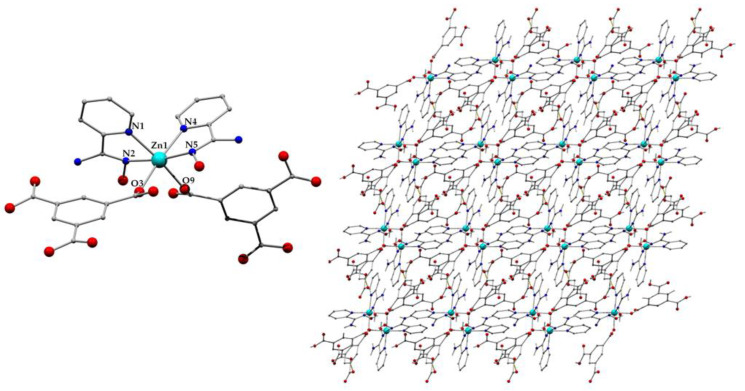
Representation of the molecular structure of compound **1** (**left**) and the 3D network that is formed through intermolecular interactions along *c* axis (**right**). Colour code: Zn^II^, cyan; N, blue; O, red; C, grey.

**Figure 2:**
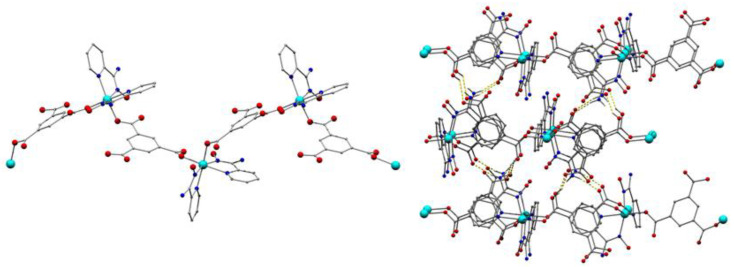
Representation of a part of the 1D chain in **2** (**left**) and the 3D network that is formed through intermolecular interactions (**right**). Colour code: Zn^II^, cyan; N, blue; O, red; C, grey.

**Figure 3:**
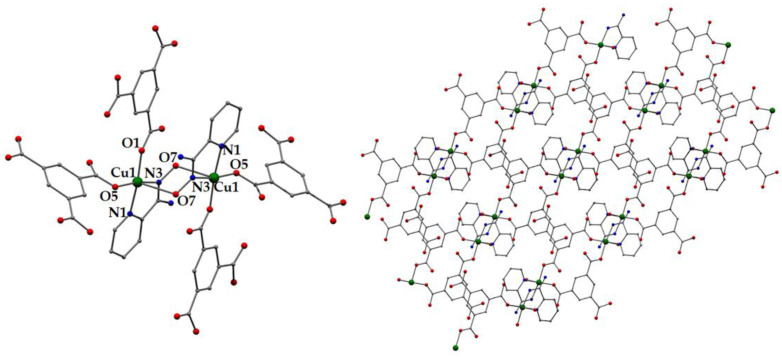
Representation of the repeating unit (**left**) and a part of the 2D network of **3** (**right**). Colour code: Cu, green; N, blue; O, red; C, grey. The hydrogen atoms are omitted for clarity.

**Figure 4:**
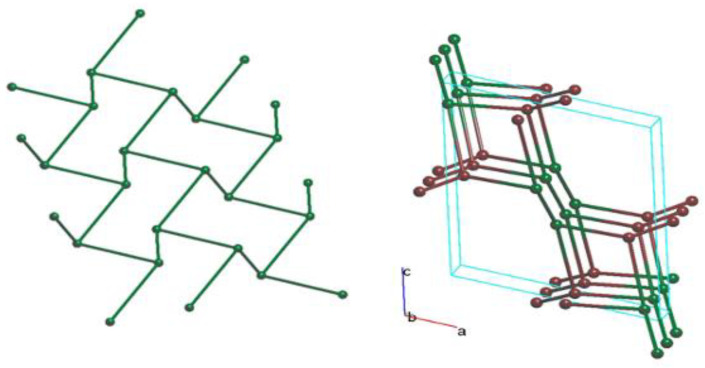
Left: representation of the underlying 3-c net with **hcb** topology in **3**. Right: representation of the 3D network with **utp** topology, which is formed when the intermolecular interactions between the carboxylate ligands are considered. The unit cell is shown with a blue line. Colour code: Cu, green; Hbtc^2−^, brown.

**Figure 5:**
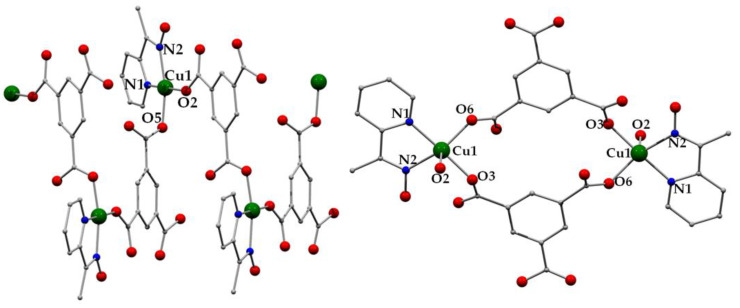
Left: representation of a part of the zig-zag chain in **4**. Right: representation of the molecular structure of **5**. Colour code: Cu^II^, green; N, navy blue; O, red; C, grey. The hydrogens are omitted for clarity.

Representations of the molecular structures of **1**–**5** are shown in Figure 1, Figure 2, Figure 3, Figure 4 and Figure 5, Figures S1 and S2 (Supplementary Information). Selected interatomic distances and angles are listed in Tables S1–S5.

Compound **1** crystallizes in the triclinic space group *P*ī. Its structure (Figure 1) consists of the mononuclear complex [Zn(H_2_btc)_2_(H_2_pyaox)_2_] and two lattice H_2_O molecules. The coordination sphere of the metal ion is completed by two neutral *N*,*N*’-bidentate chelating H_2_pyaox ligands and two monodentate single deprotonated H_2_btc^-^ ions. Zn^II^ is six coordinated displaying a slightly distorted octahedral geometry due to the relatively small bite angle of the chelating ligand (N1-Zn1-N2 = 75.1(2)° and N4-Zn1-N5 = 76.0(2)°).

There are strong intermolecular hydrogen bonding interactions in **1** that stabilize its structure and result in the formation of a three-dimensional network (Figure 1, right). In particular, there are three different types of hydrogen bonds in **1**, which involve: (1) the oxygen atoms of the oximic groups (O1 and O2), which are the donors, and the carboxylate groups of the H_2_btc^−1^ ligands (O4, O10), which act as the acceptors (O2···O4 = 2.623 Å, H2A···O4 = 1.857 Å, O2-H2A···O4 = 154.81°; O1···O10 = 2.606 Å, H1···O10 = 1.791 Å, O1-H1···O10 = 172.25°); (2) the oxygen atoms of the neutral carboxylic groups of the H_2_btc^−1^ ligand (O11, O7; donors) and the deprotonated carboxylate groups (O5, O12; acceptors) of neighbouring species (O11···O5 = 2.645 Å, H11···O5 = 1.86 Å, O11-H11···O5 = 160.15°; O7···O12 = 2.705 Å, H7···O12 = 1.942 Å, O7-H7···O12 = 154.42°) and (3) the amino group of the oximic ligand (N3; donor) and the carboxylate (O8; acceptor) group from a neighbouring complex (N3···O8 = 3.022 Å, H3B···O8 = 2.433 Å, N3-H3B···O8 = 126.27°). Furthermore, the lattice H_2_O molecules (O15, O16) form hydrogen bonds with the carboxylic (O3, O6, O13 and O4) and the oximic (O1) groups; the non-location of the lattice H_2_O hydrogen atoms precludes a detailed description of the latter.

**2** crystallizes in the chiral orthorhombic space group *P2_1_2_1_2_1_*; its structure contains a one-dimensional chain based on the repeating unit [Zn(Hbtc)(H_2_pyaox)_2_]. The metal centre is linked to two neutral *N,N*’-bidentate chelating H_2_pyaox ligands, and two terminally ligated carboxylate groups coming from two different Hbtc^2−^ ions; the latter bridges two neighbouring repeating units adopting an *η*^1^:*η*^1^:*μ* coordination mode. Zn^II^ is six-coordinate adopting an octahedral geometry. The repeating unit in **2** is similar to compound **1**, with the only difference being the protonation level and, hence, the bridging capability of the carboxylate ligand (**1**, single deprotonated; **2**, double deprotonated carboxylate). The 1D chains in **2** interact strongly through hydrogen bonds, which result in the formation of a 3D network (Figure 2, right). The hydrogen bonds involve the oximic, amino and carboxylic groups as donors and the deprotonated carboxylate groups of the Hbtc^2^- ligands as acceptors. The metric parameters of the crystallographically established, independent hydrogen bonds are listed in Table S6 in the Supplementary Material.

Compound **3** crystallizes in the monoclinic space group *P2_1_/n*. Its structure consists of a two-dimensional network based on the dinuclear centrosymmetric repeating unit [Cu_2_(Hbtc)_2_(H_2_pyaox)_2_] (Figure 3). The two metal ions within the repeating unit are held together through the two bridging H_2_pyaox ligands, and their distance was 4.397 Å. The coordination sphere of Cu^II^ was completed by two Hbtc^2−^ ions, which also linked the neighbouring SBUs resulting in the formation of a 2D framework. The metal ions were penta-coordinated and adopted a square pyramidal geometry (τ = 0.05) with O7 from the oximic group occupying the apical position [78].

Intrachain hydrogen bonds stabilized the crystal structure of **3**; these were formed between: (1) the oximic group (O7), which is the donor, and the carboxylic group of the Hbtc^2−^ ion (O2), which acts as the acceptor (O7···O2 = 2.525 Å, H1(O7)···O2 = 1.664 Å, O7-H1(O7)···O2 = 173.62°), and (2) the amino group (N2), which is the donor, and the carboxylic group of the Hbtc^2−^ ion (O6), which acts as the acceptor (N2···O6 = 2.958 Å, H2(N2)···O6 = 2.202 Å, N2-H2(O6)···O6 = 147.30°). Furthermore, interchain hydrogen bonds are formed between the oxygen atoms of neighbouring carboxylic groups of the Hbtc^2−^ ions; (O4) acts as a donor and (O6) acts as an acceptor (O4···O6 = 2.64 Å, H1(O4)···O6 = 1.81 Å, O4-H1(O4)···O6 = 159.27°), forming a three dimensional network (Figure S1).

The framework in **3** forms a 3-c uninodal net [79,80,81] of **hcb** topology (Figure 4, left) with the layers being parallel to the (1,0,1) plane [82,83,84]. Taking also into account the intermolecular interactions between the Hbtc^2−^ ligands, the resulted 3D framework exhibited a **utp** topological type (Figure 4, right) [85,86,87]. Thermal stability studies in **3** revealed that it remains stable until 320 °C (Figure S2). In particular, there is a small mass loss (<5%) between room temperature and 320 °C; a sharp mass loss (*ca.* 60%) is then observed, and the decomposition of the compound continued at a steady rate with a further mass decrease of 12% between 320 and 600 °C.

**4** crystallized in the orthorhombic space group *Pna2_1_*; it is a zig-zag chain (Figure 5, left), formed by the connection of the [Cu(Hbtc)(Hmpko)] repeating units through the *η*^1^: *η*^1^:*μ* Hbtc^2−^ ligands. The coordination sphere of Cu^II^ was completed by one neutral *N,N’*-bidentate chelating Hmpko ligand. Cu^II^ was the tetra-coordinate with a square planar geometry (N1-Cu1-O2 = 173.56°). A strong intramolecular hydrogen bonding interaction was formed between the neutral oximic group (O1, donor) and a carboxylate group (O3, acceptor) from the Hbtc^2−^ ligand (O1···O3 = 2.516 Å, H1O1···O3 = 1.7 Å, O1-H1O1···O3 = 173.11°). 

Compound **5**•4H_2_O crystallized in the triclinic space group *P*ī. Its structure consisted of centrosymmetric dinuclear [Cu_2_(Hbtc)_2_(Hmpko)_2_(H_2_O)_2_] species (Figure 5, right) and H_2_O lattice molecules. The two metal centres were held together through the *η*^1^:*η*^1^:*μ* Hbtc^2−^ ions. The coordination sphere of each Cu^II^ was completed by an *N,N’*-bidentate chelating Hmpko ligand and one terminal H_2_O molecule. Each cation was penta-coordinated adopting a distorted square pyramidal geometry (τ = 0.30) with the O2 from the terminally ligated H_2_O to occupy the axial position [78]. There was a strong network of hydrogen bonding interactions that stabilized the structure of **5**•4H_2_O and result in the formation of a three-dimensional framework (Figure S3). These involve the lattice H_2_O molecules (O9 and O10), which act as both donors and acceptors, the oximic group (O1, donor), the terminally ligated H_2_O (O2, donor), the neutral carboxylic group (O8, donor) and the carboxylate groups from the Hbtc^2−^ ligands (O4, O5) that act as acceptors. The metric parameters of the crystallographically established, independent hydrogen bonds are listed in Table S7 in the Supplementary Material. The aromatic rings of the oximic and carboxylic ligands of neighbouring dimers in **5**•4H_2_O interact further through strong π–π stacking interactions, with the distance between the centroids being 3.8 Å (Figure S4).

**1–5** belong to a new family of oximic metal compounds, coordination polymers and MOFs with **3** being the first MOF based on 2-pyridyl amidoxime and 1,3,5-benzenetricarboxylic acid; furthermore, it is only the third example of a MOF based on a 2-pyridyl oxime, in general [66]. **2** and **4** join a small family of coordination polymers containing an oximic ligand [66,67]. The structures of all the reported compounds are stabilized through strong intermolecular interaction, forming three dimensional networks. The purity and stability of **1–5** has been verified by pxrd studies (Figures S5 and S6).

### 2.3. Magnetism Studies

**Figure 6:**
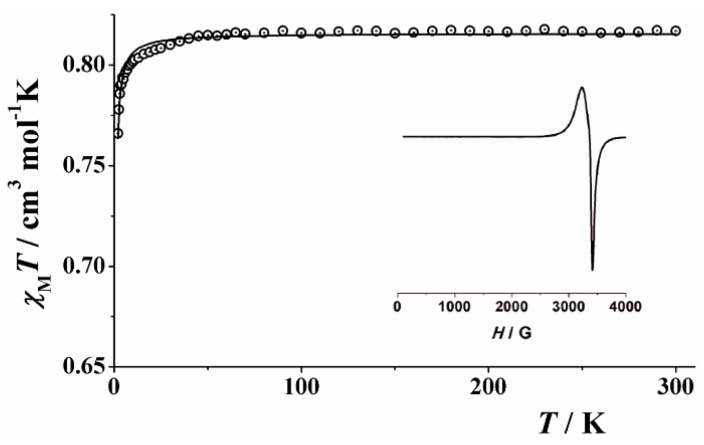
Representation of the *χ*_M_*T* as a function of *T* for **3**. Solid line represents the best fit. Inset, EPR spectrum measured at X-band frequency.

Dc magnetic susceptibility measurements were carried out on a powdered and pressed sample of **3** in the 2–300 K temperature range and under a field of 0.03 T and plotted as *χ*_M_*T* vs. *T* plot. (Figure 6). The *χ*_M_*T* for **3** was almost constant with a room temperature value of 0.8 cm^3^·Kmol^−1^ in good agreement with two non-interacting Cu(II) cations (0.375 cm^3^·Kmol^−1^) with an overall *g* greater than 2.00.

The fitting of the experimental data was performed by using the spin-only Hamiltonian *H = −2J(Ŝ_1_·Ŝ_2_)* and PHI software [88] and resulted in almost non-interacting Cu(II) ions with a *J* = −0.16(1) cm^−1^ and a global *g* value of 2.085(1).

This almost irrelevant value of the superexchange coupling *J* is expected due to the relative arrangement of the Cu(II) cations, which determines the overlap along the long bond distance involving the non-magnetic dz^2^ orbital and also due the position of the oximes ligands, where the Cu(II)-O-N-Cu(II) torsion angle was 86.8° close to orthogonality. The almost isotropic value of *g* was confirmed by electronic paramagnetic resonance (EPR) spectroscopy at X-band frequency, which provides the low temperature *g*_||_ and g_ꓕ_ values of 2.16 and 2.08 (Figure 6, inset), being in total agreement with the dc measurements.

### 2.4. Reactivity Studies

The existence of free (non-coordinated) NH_2_- groups in the crystal structure of **3** prompted us to study the ability of this MOF to adsorb or react with metal ions in the aqueous environment. The reactivity studies were carried out by soaking crystals of **3** into solutions of 0.10–0.30 mmol of metal salts (Fe(NO_3_)_3_, Ni(NO_3_)_2_, Co(NO_3_)_2_ and CrCl_3_) in H_2_O (10 mL). The MOF crystals were activated prior to the metal ion encapsulation to remove the amount of solvent present; this was carried out by stirring the crystals in DMF for several hours and then exchanging this solvent with acetone, which is easily removed at 80 °C. The metal encapsulation was initially investigated by batch studies using UV–vis spectroscopy (Figures S7 and S8). The UV-vis studies for Fe(NO_3_)_3_ revealed a substantial decrease of the concentration of the metal ion in the solution over the first 4 min, which was then followed by the appearance of an additional peak at 290 nm (Figure S5); the latter can be potentially attributed to Cu^2+^ ions in the solution [89], which indicates that a chemical change takes place in the structure of **3** upon reaction with other metal ions. The progress of the reaction was investigated in the case of Fe(NO_3_)_3_ by means of FTIR and single-crystal X-ray crystallography. During the reaction, the green crystals of **3** had been replaced by a brown precipitate (Figure S9) and the initial yellow colour of the solution had turned to pale green. The solution was then filtered and left in a closed vial at room temperature for one day, after which a few green-cyan crystals were formed; the latter were characterized with IR spectroscopy and single crystal X-ray diffraction (unit cell comparison) and were found to be the mononuclear complex [Cu(H_2_pyca)_2_(H_2_O)]·(NO_3_)_2_, where H_2_pyca = pyridine-2-carboxamide [90]. H_2_pyca is the product of the hydrolysis of H_2_pyaox, which is a reaction often encountered in oximes [52,91]. Concerning the brown precipitate, this was amorphous, which prevented from its further; it is worth to mention, though, that the IR spectrum (Figure S10) of the brown precipitate indicates that this could correspond to the previously reported 1D coordination polymer [Fe_3_(H_2_O)_12_(btc)_2_]_n_ [76]. The absence of bands in the range 1730–1690 cm^−1^ reveals the complete deprotonation of the organic ligand in the product of the reaction.

## 3. Materials and Methods 

### 3.1. Materials, Physical and Spectroscopic Measurements

All the manipulations were performed under aerobic conditions using materials (reagent grade) and solvents as received. Hmpko and H_2_pyaox were prepared as described elsewhere [92,93]. Warning: Perchlorate salts are potentially explosive; such compounds should be used in small quantities and treated with utmost care at all times. 

Elemental analysis (C, H and N) was performed by the in-house facilities of National University of Ireland Galway, School of Chemistry. IR spectra (4000–400 cm^−1^) were recorded on a Perkin-Elmer Spectrum 400 FT-IR spectrometer. Powder X-ray diffraction data (pxrd) were collected using an Inex Equinoz 6000 diffractometer. Solid TGA experiments were performed on a STA625 thermal analyser from Rheometric Scientific (Piscataway, NJ, USA). The heating rate was kept constant at 10 °C/min, and all runs were carried out between 20 and 600 °C. The measurements were made in open aluminium crucibles, nitrogen was purged in ambient mode and calibration was performed using an indium standard. Solid-state, variable-temperature and variable-field magnetic data were collected on powdered samples using an MPMS5 Quantum Design magnetometer operating at 0.03 T in the 300–2.0 K range for the magnetic susceptibility and at 2.0 K in the 0–5 T range for the magnetization measurements. Diamagnetic corrections were applied to the observed susceptibilities using Pascal’s constants. EPR spectrum was collected using a Bruker 300 spectrometer with an X-band frequency measured at room temperature.

### 3.2. Compound Synthesis

#### 3.2.1. Synthesis of [Zn(H_2_btc)_2_(H_2_pyaox)_2_] 2H_2_O (1•2H_2_O)

Zn(ClO_4_)_2_•6H_2_O (0.037 g, 0.10 mmol) and H_2_pyaox (0.027g, 0.20 mmol) were dissolved in H_2_O (10 mL). The resultant solution was put in the oven and heated at 100 °C for 1 h. Then, H_3_btc (0.021 g, 0.1 mmol) was added and the vial was placed into the oven for 24 h, after which X-ray quality colourless crystal needles of **1**•2H_2_O were formed. The crystals were collected by filtration, washed with cold MeCN (2 mL) and Et_2_O (2 × 5 mL) and dried in air. Yield 55 %. Anal. Calc. for **1**•2H_2_O: C, 45.38; H, 3.55; N, 10.58. Found: C, 45.87; H, 3.71; N, 10.09 %. IR data: ν (cm^−1^) = 3484 m, 3406 m, 3310 m, 2982 m, 2757 m, 2564 m, 2364 w, 1943 w, 1724 m, 1698 m, 1670 m, 1598 s, 1574 m, 1543 s, 1497 m, 1406 m, 1369 m, 1281 m, 1247 m, 1224 s, 1177 s, 1153 m, 1096 m, 1022 s, 907 m, 844 m, 790 s, 745 s, 691 s, 665 m.

#### 3.2.2. Synthesis of [Zn(Hbtc)(H_2_pyaox)_2_]_n_ (**2**)

Zn(O_2_CMe)_2_•6H_2_O (0.022 g, 0.10 mmol), H_2_pyaox (0.027 g, 0.20 mmol) and Et_3_N (56 μL, 0.4 mmol) were dissolved in H_2_O (10 mL). The resultant solution was put in the oven and heated at 100 °C for 1h. H_3_btc (0.021 g, 0.10 mmol) was added and the vial was placed into the oven for 24 h, after which X-ray quality colourless needless of **2** were formed. The crystals were collected by filtration, washed with cold MeCN (2 mL) and Et_2_O (2 × 5 mL), and dried in air. Yield 70%. Anal. Calc. for **2**: C, 46.05; H, 3.31; N, 15.34. Found: C, 45.55; H, 3.63; N, 15.18%. IR data: ν (cm^−1^) = 3474 m, 3357 m, 3307 m, 3187 m, 2982 m, 1722 s, 1678 s, 1604 s, 1575 m, 1528 s, 1434 m, 1406 m, 1368 s, 1302 m, 1229 m, 1169 s, 1097 m, 1020 s, 1007 m, 1020 s, 937 m, 896 m, 855 m, 811 m, 790 s, 756 m, 714 s, 687 m, 668 s.

#### 3.2.3. Synthesis of [Cu(Hbtc)(H_2_pyaox)]_n_ (**3**)

Cu(ClO_4_)_2_•6H_2_O (0.037 g, 0.10 mmol) and H_2_pyaox (0.027 g, 0.20 mmol) were dissolved in H_2_O (10 mL). The resultant solution was put in the oven and heated at 100 °C for 1 h. The colour of the solution turned cyan and H_3_btc (0.021 g, 0.1 mmol) was then added; the vial was placed into the oven for 24 h, after which X-ray quality green crystals of **3** were formed. The crystals were collected by filtration, washed with cold MeCN (2 mL) and Et_2_O (2 × 5 mL), and dried in air. Yield 85%. Anal. Calc. for **3**: C, 44.07; H, 2.71; N, 10.28. Found: C, 44.32; H, 3.07; N, 10.21 %. IR data: *ν* (cm^−1^) = 3443 w, 3314 w, 1726 m, 1682 m, 1612 s, 1579 w, 1544 s, 1492 w, 1437 w, 1421 w, 1364 m, 1296 w, 1274 w, 1242m, 1238 m, 1179 m, 1157 w, 1095 m, 1036 s, 951 m, 928 w, 895 w, 836 m, 805 w, 786 s, 743 s, 720 s, 689 w, 669 m.

#### 3.2.4. Synthesis of [Cu(Hbtc)(HmpKo)]_n_ (**4**) 

Cu(ClO_4_)_2_•6H_2_O (0.037 g, 0.10 mmol) and HmpKo (0.014 g, 0.10 mmol) were dissolved in H_2_O (5 mL). The resultant solution was put in the oven and heated at 100 °C for 1 h. H_3_btc (0.021 g, 0.1 mmol) was then added and the vial was placed into the oven for 24 h, after which X-ray quality blue crystals of **5** were formed. The crystals were collected by filtration, washed with cold MeCN (2 mL) and Et_2_O (2 × 5 mL), and dried in air. Yield 73%. Anal. Calc. for **4**: C, 47.12; H, 2.97; N, 6.87. Found: C, 46.81; H, 3.14; N, 7.01 %. IR data: *ν* (cm^−1^) = 2247 w, 2167 w, 1813 m, 1720 m, 1606 m, 1549 s, 1482 w, 1424 m, 1360 s, 1299 w, 1266 w, 1244 s, 1177 s, 1160 s, 1149 w, 1100 w, 1085 m, 1053 w, 1027 w, 978 w, 957 m, 923 m, 803 w, 770 m, 749 s, 718 s, 690 m, 673 s.

#### 3.2.5. Synthesis of [Cu_2_(Hbtc)_2_(Hmpko)_2_(H_2_O)_2_] 4H_2_O (**5**•4H_2_O)

Cu(NO_3_)_2_•2.5H_2_O (0.093 g, 0.40 mmol) and Hmpko (0.014 g, 0.10 mmol) were dissolved in in H_2_O (10 mL). The resultant solution was put in the oven and heated at 100 °C for 1 h. Then, H_3_btc (0.021 g, 0.1 mmol) was added and the vial was placed into the oven for 24 h, after which X-ray quality cyan crystals of **5**•4H_2_O were formed. The crystals were collected by filtration, washed with cold MeCN (2ml) and Et_2_O (2 × 5 mL), and dried in air. Yield 40%. Anal. Calc. for **5**•4H_2_O: C, 41.61; H, 3.93; N, 6.07. Found: C, 41.29; H, 4.09; N, 6.34 %. IR data: *ν* (cm^−1^) = 3393 w, 3032 w, 1714 m, 1606 s, 1548 s, 1435 s, 1363 s, 1335 w, 1249 m, 1235 m, 1189 m, 1144 m, 1103 m, 1074 m, 1028 w, 930 m, 855 w, 803 w, 778 s, 744 s, 714 s, 677 s.

### 3.3. Single-Crystal X-ray Crystallography

Single crystal diffraction data for **1–5** were collected in an Oxford Diffraction Xcalibur CCD diffractometer using graphite-monochromatic Mo Ka radiation (λ = 0.71073 Å) at room temperature. The structures were solved using SHELXT [94], embedded in the OSCAIL software [95]. The non-H atoms were treated anisotropically, whereas the hydrogen atoms were placed in calculated, ideal positions and refined as riding on their respective carbon atoms. The hydrogen atoms on water molecules cannot be calculated accurately and are best located in difference maps and then refined. In the case of compound **1**•2H_2_O, it was not possible to locate the water H atoms in difference maps. Molecular graphics were produced with DIAMOND [96].

Unit cell data and structure refinement details are listed in Table 1. CIF files can be obtained free of charge at www.ccdc.camac.uk/retrieving.html or from the Cambridge Crystallographic Data Centre, Cambridge, UK with the REF codes 2,054,509–2,054,513 for **1**–**5**.

## 4. Conclusions

The employment of 2-pyridyl oximes (pyridine−2 amidoxime, H_2_pyaox; 2-methyl pyridyl ketoxime and Hmpko) in combination with 1,3,5-benzenetricarboxylic acid (H_3_btc) provided access to five new compounds, including discrete clusters, coordination polymers and MOFs. Among them, [Cu(Hbtc)(H_2_pyaox)]_n_ (**3**) was based on a 3-c uninodal net of **hcb** topology being the first reported MOF bearing H_2_pyaox; it was also the third MOF example based on a 2-pyridyl oxime in general. [Zn(Hbtc)(H_2_pyaox)_2_]_n_ (**2**) and [Cu(Hbtc)(HmpKo)]_n_ (**4**) joined a small family of coordination polymers containing an oximic ligand. **1**–**5** formed a 3D supramolecular network through strong hydrogen bonding interactions.

The exchange interactions between the metal centres in **3** were investigated through dc magnetic susceptibility measurements and were found to be very weak antiferromagnetic (*J* = −0.16(1) cm^−1^). Finally, reactivity studies were performed for **3** in the presence of metal ions; in the case of Fe(NO_3_)_3_, the reaction products were [Cu(H_2_pyca)_2_(H_2_O)]·(NO_3_)_2_, (H_2_pyca = pyridine-2-carboxamide, coming from the hydrolysis of the oximic ligand) and [Fe_3_(H_2_O)_12_(btc)_2_]_n_. The reaction products were characterized by single crystal X-ray crystallography and IR spectroscopic techniques.

## Figures and Tables

**Table 1:** Crystallographic data for complexes **1**–**5**.

Complex	1.2H_2_O	2	3
Empirical formula	C_30_H_23_N_6_O_16_Zn	C_21_H_18_N_6_O_8_Zn	C_15_H_11_CuN_3_O_7_
Formula weight	788.91	547.78	408.81
Crystal system	Triclinic	Orthorhombic	Monoclinic
Space group	*P*ī	*P2_1_2_1_2_1_*	*P2_1_/n*
*a* (Å)	10.4432(8)	8.9165(2)	13.6020(5)
*b* (Å)	11.0391(10)	14.3950(4)	7.8643(3)
*c* (Å)	16.1331(10)	18.0662(4)	14.5936(6)
*α* (^o^)	70.263(7)	90	90
*β* (^o^)	71.936(6)	90	106.629(4)
*γ* (^o^)	74.980(7)	90	90
*V* (Å^3^)	1639.4(2)	2318.85(10)	1495.79(10)
*Ζ*	2	4	4
*ρ*_calc_ (g cm^−3^)	1.598	1.569	1.815
μ (mm^−1^)	0.837	1.120	1.510
Measured/independent reflections (*R*_int_)	12,784/7550 (0.0634)	19,382/ 5593 (0.0303)	10,552/3386 (0.0279)
Parameters refined	484	350	251
GoF (on *F*^2^)	0.972	1.043	1.069
*R*_1_^a^ (*I* > 2*σ*(*Ι*))	0.0948	0.0340	0.0379
*wR*_2_^b^ (*I* > 2*σ*(*Ι*))	0.2333	0.0763	0.1025
(*Δρ*)_max_/(*Δρ*)_min_ (e Å^−3^)	2.739/−0.619	0.879/−0.338	0.539/−0.683
Complex	**4**	**5**•4H_2_O	
Empirical formula	C_21_H_11_CuN_2_O_7_	C_16_H_18_CuN_2_O_10_	
Formula weight	466.86	461.86	
Crystal system	Orthorhombic	Triclinic	
Space group	*Pna2_1_*	*P*ī	
*a* (Å)	14.4904(8)	8.5820(6)	
*b* (Å)	14.2054(6)	10.7135(8)	
*c* (Å)	7.4068(5)	11.0721(8)	
*a* (^o^)	90	64.108(7)	
*β* (^o^)	90	86.688(6)	
*γ* (^°^)	90	83.015(6)	
*V* (Å^3^)	2.034	909.01(12)	
*Ζ*	4	2	
*ρ*_calc_ (g cm^−3^)	2.034	1.687	
μ (mm^−1^)	1.494	1.263	
Measured/independent reflections (*R*_int_)	12,706/3688 (0.0784)	8056/4204 (0.0377)	
Parameters refined	237	286	
GoF (on *F*^2^)	0.974	0.963	
*R*_1_^a^ (*I* > 2*σ*(*Ι*))	0.0544	0.0417	
*wR*_2_^b^ (*I* > 2*σ*(*Ι*))	0.0869	0.0935	
(*Δρ*)_max_/(*Δρ*)_min_ (e Å^−3^)	0.420/−0.405	0.496/−0.489	

^a^*R*_1_ = Σ(|*F*_o_|−|*F*_c_|)/Σ(|*F*_o_|); ^b^ *wR*_2_ = {Σ[*w*(*F*_o_^2^−*F*_c_^2^)^2^]/Σ[*w*(*F*_o_^2^)^2^]}^1/2^

## Supplementary Materials

The following are available online, Figure S1: Representation of the 3D network formed through hydrogen bonding interactions in **3**, Figure S2: Representation of the 3D network formed through hydrogen bonding interactions in **5**•4H_2_O, Figure S3: Comparison of the pxrd patterns for **3** (theoretical, red; experimental: green; CH_2_Cl_2_, navy blue; EtOH, grey; H_2_O, magenta; Me_2_CO, cyan; MeOH, pink, Figure S4: Comparison of the pxrd patterns for **5**•4H_2_O (theoretical, red; experimental, blue), Figure S5: UV-vis plot for the adsorption of iron(III) nitrate nonahydrate by **3** in H_2_O, Figure S6: Photo of crystals of **3** (left) before the reaction and the formed brown compound (right), Figure S7: The infrared spectra of the isolated brown precipitated, Table S1: Selected interatomic distances (Å) and angles for **1**•2H_2_O, Table S2: Selected interatomic distances (Å) and angles for **2**, Table S3: Selected interatomic distances (Å) and angles for **3**, Table S4: Selected interatomic distances (Å) and angles for **4**, Table S5: Selected interatomic distances (Å) and angles for **5**•4H_2_O, Table S6: Hydrogen bonding details for **2**, Table S7: Hydrogen bonding details for **5**•4H_2_O.

## Sample Availability

Samples of the compounds **1**–**5** are available from the authors.

## References

[1] Qin J.-S., Du D.-Y., Guan W., Bo X.-J., Li Y.-F., Guo L.-P., Su Z.-M., Wang Y.-Y., Lan Y.-Q., Zhou H.-C. (2015). Ultrastable Polymolybdate-Based Metal–Organic Frameworks as Highly Active Electrocatalysts for Hydrogen Generation from Water. J. Am. Chem. Soc..

[2] Kuznetsova A., Matveevskaya V., Pavlov D., Yakunenkov A., Potapov A. (2020). Coordination Polymers Based on Highly Emissive Ligands: Synthesis and Functional Properties. Materials.

[3] Hu Z., Deibert B.J., Li J. (2014). Luminescmakeent metal–organic frameworks for chemical sensing and explosive detection. Chem. Soc. Rev..

[4] Douvali A., Tsipis A.C., Eliseeva S.V., Petoud S., Papaefstathiou G.S., Malliakas C.D., Papadas I., Armatas G.S., Margiolaki I., Kanatzidis M.G. (2015). Turn-on luminescence sensing and real-time detection of traces of water in organic solvents by a flexible metal-organic framework. Angew. Chem. Int. Ed..

[5] Ma L., Falkowski J.M., Abney C., Lin W. (2010). A series of isoreticular chiral metal–organic frameworks as a tunable platform for asymmetric catalysis. Nat. Chem..

[6] Wu M.-X., Yang Y.-W. (2017). Metal–Organic Framework (MOF)-Based Drug/Cargo Delivery and Cancer Therapy. Adv. Mater..

[7] Huxford R.C., Rocca J.D., Lin W. (2010). Metal-organic frameworks as potential drug carriers. Curr. Opin. Chem. Biol..

[8] Yan S., Feng L., Wang K., Pang J., Bosch M., Lollar C., Sun Y., Qin J., Wang X., Zhang P. (2018). Stable Metal–Organic Frameworks: Design, Synthesis, and Applications. Adv. Mater..

[9] Zhou H.-C., Kitagawa S. (2014). Metal-Organic Frameworks (MOFs). Chem. Soc. Rev..

[10] Eddaoudi M., Moler D.B., Li H., Chen B., Rheineke T.M., O’Keefe M., Yaghi O.M. (2001). Modular Chemistry:  Secondary Building Units as a Basis for the Design of Highly Porous and Robust Metal−Organic Carboxylate Frameworks. Acc. Chem. Res..

[11] Miyasaka H., Julve M., Yamashita M., Clérac R. (2009). Slow Dynamics of the Magnetization in One-Dimensional Coordination Polymers: Single-Chain Magnets. Inorg. Chem..

[12] Coulon C., Miyasaka H., Clérac R. (2006). Single-Chain Magnets: Theoretical Approach and Experimental Systems. Struct. Bonding.

[13] Gatteschi D., Sessoli R. (2003). Quantum Tunneling of Magnetization and Related Phenomena in Molecular Materials. Angew. Chem., Int. Ed..

[14] Givaja G., Amo-Ochoa P., Gomez-Garcia C., Zamora F. (2012). Electrical conductive coordination polymers. Chem. Soc. Rev..

[15] Yue Q., Gao E.-Q. (2019). Azide and carboxylate as simultaneous coupler for magnetic coordination polymers. Coord. Chem. Rev..

[16] Leuenberger M.N., Loss D. (2001). Quantum computing in molecular magnets. Nature.

[17] Bogani L., Wernsdorfer W. (2008). Molecular spintronics using single-molecule magnets. Nat. Mater..

[18] Papatriantafyllopoulou C., Zartilas S., Manos M.J., Pichon C., Clérac R., Tasiopoulos A.J. (2014). A single-chain magnet based on linear [Mn^III^_2_Mn^II^] units. Chem. Commun..

[19] Hui J., Kishida H., Ishiba K., Takemasu K., Morikawa M., Kimizuka N. (2016). Ferroelectric Coordination Polymers Self-Assembled from Mesogenic Zinc(II) Porphyrin and Dipolar Bridging Ligands. Chem. Eur. J..

[20] Chen L., Ji Q., Wang X., Pan Q., Ccao X., Xu G. (2017). Two novel metal–organic coordination polymers based on ligand 1,4-diazabicyclo[2.2.2]octane N,N′-dioxide with phase transition, and ferroelectric and dielectric properties. Cryst. Eng. Comm..

[21] Wang H.-N., Meng X., Dong L.-Z., Chen Y., Li S.-L., Lan Y.-Q. (2019). Coordination polymer-based conductive materials: Ionic conductivity vs. electronic conductivity**. J. Mater. Chem. A.

[22] Jeon I.-R., Clérac R. (2012). Controlled association of single-molecule magnets (SMMs) into coordination networks: Towards a new generation of magnetic materials. Dalton Trans..

[23] Bernot K., Luzon J., Sessoli R., Vindigni A., Thion J., Richeter S., Leclercq D., Larionova J., Van der Lee A. (2008). The Canted Antiferromagnetic Approach to Single-Chain Magnets. J. Am. Chem. Soc..

[24] Wang T.-T., Ren M., Bao S.-S., Liu B., Pi L., Cai Z.-S., Zheng Z.-H., Xu Z.-L., Zheng L.-M. (2014). Effect of Structural Isomerism on Magnetic Dynamics: From Single-Molecule Magnet to Single-Chain Magnet. Inorg. Chem..

[25] Manos M.J., Markoulides M.S., Malliakas C.D., Papaefstathiou G.S., Chronakis N., Kanatzidis M.G., Trikalitis P.N., Tasiopoulos A.J. (2011). A Highly Porous Interpenetrated Metal–Organic Framework from the Use of a Novel Nanosized Organic Linker. Inorg. Chem..

[26] Ugale B., Singh Dhankhar S., Nagaraja C.M. (2017). Interpenetrated Metal–Organic Frameworks of Cobalt(II): Structural Diversity, Selective Capture, and Conversion of CO_2_. Cryst. Growth Des..

[27] Sezginel K.B., Feng T., Wilmer C.E. (2017). Discovery of hypothetical hetero-interpenetrated MOFs with arbitrarily dissimilar topologies and unit cell shapes. Cryst. Eng. Comm..

[28] Nouar F., Devic T., Chevreau H., Guillou N., Gibson E., Clet G., Daturi M., Vimont A., Grenèche J.M., Breeze M.I. (2012). Tuning the breathing behaviour of MIL-53 by cation mixing. Chem. Commun..

[29] Alhamami M., Doan H., Cheng C.-H. (2014). A Review on Breathing Behaviors of Metal-Organic-Frameworks (MOFs) for Gas Adsorption. Materials.

[30] Kou W.-T., Yang C.-X., Yan X.-P. (2018). Post-synthetic modification of metal–organic frameworks for chiral gas chromatography. J. Mater. Chem. A.

[31] Wang Z., Li Z., Ng M., Milner P.J. (2020). Rapid mechanochemical synthesis of metal–organic frameworks using exogenous organic base. Dalton Trans..

[32] Klinowski J., Almeida Paz F.A., Silva P., Rocha J. (2011). Microwave-Assisted Synthesis of Metal–Organic Frameworks. Dalton Trans..

[33] Vinu M., Lin W.-C., Senthil Raja D., Han J.-L., Lin C.-H. (2017). Microwave-Assisted Synthesis of Nanoporous Aluminum-Based Coordination Polymers as Catalysts for Selective Sulfoxidation Reaction. Polymers.

[34] Garibay S.J., Cohen S.M. (2010). Isoreticular synthesis and modification of frameworks with the UiO-66 topology. Chem. Commun..

[35] Yaghi O.M., O’Keeffe M., Ockwig N.W., Chae H.K., Eddaoudi M., Kim J. (2003). Reticular synthesis and the design of new materials. Nature.

[36] Ardila-Suárez C., Díaz-Lasprilla A.M., Díaz-Vaca L.A., Balbuena P.B., Baldovino-Medrano V.G., Ramírez-Caballero G.E. (2019). Synthesis, characterization, and post-synthetic modification of a micro/mesoporous zirconium–tricarboxylate metal–organic framework: Towards the addition of acid active sites. Cryst. Eng. Comm..

[37] Chen D., Zhao J., Zhang P., Dai S. (2019). Mechanochemical synthesis of metal–organic frameworks. Polyhedron.

[38] Mai Z., Liu D. (2019). Synthesis and Applications of Isoreticular Metal–Organic Frameworks IRMOFs-n (n = 1, 3, 6, 8). Cryst. Growth Des..

[39] Horcajada P., Surblé S., Serre C., Hong D.-Y., Seo Y.-K., Chang J.-S., Grenèche J.-M., Margiolaki I., Férey G. (2007). Synthesis and catalytic properties of MIL-100(Fe), an iron(iii) carboxylate with large pores. Chem. Commun..

[40] Kourtellaris A., Moushi E.E., Spanopoulos I., Tampaxis C., Charalambopoulou G., Steriotis T.A., Papaefstathiou G.S., Trikalitis P.N., Tasiopoulos A.J. (2016). A microporous Cu^2+^ MOF based on a pyridyl isophthalic acid Schiff base ligand with high CO_2_ uptake. Inorg. Chem. Front..

[41] Moushi E.E., Kourtellaris A., Spanopoulos I., Manos M.J., Papaefstathiou G.S., Trikalitis P.N., Tasiopoulos A.J. (2015). A Microporous Co^2+^ Metal Organic Framework with Single-Crystal to Single-Crystal Transformation Properties and High CO_2_ Uptake. Cryst. Growth Des..

[42] Manos M.J., Moushi E.E., Papaefstathiou G.S., Tasiopoulos A.J. (2012). New Zn^2+^ Metal Organic Frameworks with Unique Network Topologies from the Combination of Trimesic Acid and Amino-Alcohols. Cryst. Growth Des..

[43] Clausen H.F., Poulsen R.D., Bond A.D., Chevallier M.-A.S., Iversen B.B. (2005). Solvothermal synthesis of new metal organic framework structures in the zinc terephthalic acid dimethyl formamide system. J. Solid State.

[44] Chen Z., Adil K., Weselinski L.J., Belmabkhout Y., Eddaoudi M. (2015). A supermolecular building layer approach for gas separation and storage applications: The eea and rtl MOF platforms for CO2 capture and hydrocarbon separation. J. Mater. Chem. A.

[45] Tranchemontagne D.J., Hunt J.R., Yaghi O.M. (2008). Room temperature synthesis of metal-organic frameworks: MOF-5, MOF-74, MOF-177, MOF-199, and IRMOF-0. Tetrahedron.

[46] Li H., Eddaoudi M., O’Keeffe M., Yaghi O.M. (1999). Design and synthesis of an exceptionally stable and highly porous metal-organic framework. Nature.

[47] Sapchenko S.A., Dybtsev D.N., Damsonenko D.G., Fedin V.P. (2010). Synthesis, crystal structures, luminescent and thermal properties of two new metal–organic coordination polymers based on zinc(II) carboxylates. New J. Chem..

[48] Zhao X.-L., Sun W.-Y. (2014). The organic ligands with mixed N-/O-donors used in construction of functional metal–organic frameworks. Cryst. Eng. Comm..

[49] Guesh K., Caiuby C.A.D., Mayoral Á., Díaz-García M., Díaz I., Sanchez-Sanchez M. (2017). Sustainable Preparation of MIL-100(Fe) and Its Photocatalytic Behavior in the Degradation of Methyl Orange in Water. Growth Des..

[50] Yin Z., Zhou Y.-L., Zeng M.-H., Kurmoo M. (2015). The concept of mixed organic ligands in metal–organic frameworks: Design, tuning and functions. Dalton Trans..

[51] ZareKarizi F., Johariana M., Morsali A. (2018). Pillar-layered MOFs: Functionality, interpenetration, flexibility and applications. J. Mater. Chem. A.

[52] Milios C.J., Stamatatos T.C., Perlepes S.P. (2006). The coordination chemistry of pyridyl oximes. Polyhedron.

[53] Escuer A., Vlahopoulou G., Mautner F.A. (2011). Assembly of [Mn^II^_2_Mn^III^_2_] S = 9 Clusters via Azido Bridges: A New Single-Chain Magnet. Inorg. Chem..

[54] Mowson A.M., Nguyen T.N., Abboud K.A., Christou G. (2013). Dimeric and tetrameric supramolecular aggregates of single-molecule magnets via carboxylate substitution. Inorg. Chem..

[55] Papatriantafyllopoulou C., Jones L.F., Nguyen T.D., Matamoros-Salvador N., Cunha-Silva L., Almeida Paz F.A., Rocha J., Evangelisti M., Brechin E.K., Perlepes S.P. (2008). Using pyridine amidoximes in 3d-metal cluster chemistry: A novel ferromagnetic Ni_12_ complex from the use of pyridine-2-amidoxime. Dalton Trans..

[56] Efthymiou C.G., Cunha-Silva L., Perlepes S.P., Brechin E.K., Inglis R., Evangelisti M., Papatriantafyllopoulou C. (2016). In search of molecules displaying ferromagnetic exchange: Multiple-decker Ni_12_ and Ni_16_ complexes from the use of pyridine-2-amidoxime. Dalton Trans..

[57] Papatriantafyllopoulou C., Stamatatos T.C., Wernsdorfer W., Teat S.J., Tasiopoulos A.J., Escuer A., Perlepes S.P. (2010). Combining Azide, Carboxylate, and 2-Pyridyloximate Ligands in Transition-Metal Chemistry: Ferromagnetic Ni^II^_5_ Clusters with a Bowtie Skeleton. Inorg. Chem..

[58] Polyzou C.D., Efthymiou C.G., Escuer A., Cunha-Silva L., Papatriantafyllopoulou C., Perlepes S.P. (2013). In search of 3d/4f-metal single-molecule magnets: Nickel(II)/lanthanide(III) coordination clusters. Pure Appl. Chem..

[59] Papatriantafyllopoulou C., Stamatatos T.C., Efthymiou C.G., Cunha-Silva L., Almeida Paz F.A., Perlepes S.P., Christou G. (2010). A High-Nuclearity 3d/4f Metal Oxime Cluster: An Unusual Ni_8_Dy_8_ “Core-Shell” Complex from the Use of 2-Pyridinealdoxime. Inorg. Chem..

[60] Papatriantafyllopoulou C., Estrader M., Efthymiou C.G., Dermitzaki D., Gkotsis K., Terzis A., Diaz C., Perlepes S.P. (2009). In search for mixed transition metal/lanthanide single-molecule magnets: Synthetic routes to Ni^II^/Tb^III^ and Ni^II^/Dy^III^ clusters featuring a 2-pyridyl oximate ligand. Polyhedron.

[61] Efthymiou C.G., Mylonas-Margaritis I., Das Gupta S., Tasiopoulos A., Nastopoulos V., Christou G., Perlepes S.P., Papatriantafyllopoulou C. (2019). Synthesis and characterisation of new Ni_2_Mn, Ni_2_Mn_2_ and Mn_8_ clusters by the use of 2-pyridyl oximes. Polyhedron.

[62] Stamatatos T.C., Foguet-Albiol D., Stoumpos C.C., Raptopoulou C.P., Terzis A., Wernsdorfer W., Perlepes S.P., Christou G. (2007). New Mn_3_ structural motifs in manganese single-molecule magnetism from the use of 2-pyridyloximate ligands. Polyhedron.

[63] Ghosh T., Abboud K.A., Christou G. (2019). New Mn^II^Mn^III8^ and Mn^II^_2_Mn^III^_10_Mn^IV^_2_ clusters from the reaction of methyl 2-pyridyl ketone oxime with [Mn_12_O_12_(O_2_CR)_16_(H_2_O)_4_]. Polyhedron.

[64] Nguyen T.N., Shiddiq M., Ghosh T., Abboud K.A., Hill S., Christou G. (2015). Covalently Linked Dimer of Mn_3_ Single-Molecule Magnets and Retention of Its Structure and Quantum Properties in Solution. J. Am. Chem. Soc..

[65] Nguyen T.N., Wernsdorfer W., Shiddiq M., Abboud K.A., Hill S., Christou G. (2016). Supramolecular aggregates of single-molecule magnets: Exchange-biased quantum tunneling of magnetization in a rectangular [Mn_3_]_4_ tetramer. Chem. Sci..

[66] Mylonas-Margaritis I., Gerard A., Skordi K., Mayans J., Tasiopoulos A., McArdle P., Papatriantafyllopoulou C. (2020). From 1D Coordination Polymers to Metal Organic Frameworks by the Use of 2-Pyridyl Oximes. Materials.

[67] Mylonas-Margaritis I., Winterlich M., Efthymiou C.G., Lazarides T., McArdle P., Papatriantafyllopoulou C. (2018). New insights into oximic ligands: Synthesis and characterization of 1D chains by the use of pyridine 2-amidoxime and polycarboxylates. Polyhedron.

[68] Shen T., Liu T., Yuan Z., Cui F., Jin Y., Chen X. (2020). Cu-based metal–organic framework HKUST-1 as effective catalyst for highly sensitive determination of ascorbic acid. RSC Adv..

[69] Álvarez J.R., Sánchez-González E., Pérez E., Schneider-Revueltas E., Martínez A., Tejeda-Cruz A., Islas-Jácome A., González-Zamora E., Ibarra I.A. (2017). Structure stability of HKUST-1 towards water and ethanol and their effect on its CO_2_ capture properties. Dalton Trans..

[70] Chui S., Lo S., Charmant J., Opren G., Williams I.D. (1999). A Chemically Functionalizable Nanoporous Material [Cu_3_(TMA)_2_(H_2_O)_3_]_n_. Science.

[71] Davies K., Bourne S.A., Ohrstrom L., Oliver C.L. (2010). Anionic zinc-trimesic acid MOFs with unusual topologies: Reversible hydration studies. Dalton Trans..

[72] Larsen R.W., Wojitas L. (2017). Photo-physical studies of ruthenium(II) tris(1,10-phenanthroline) confined within a polyhedral zinc(II)-trimesic acid metal organic framework. Inorg. Chim. Acta.

[73] Rajak R., Saraf M., Mobin S.M. (2019). Robust heterostructures of a bimetallic sodium–zinc metal–organic framework and reduced graphene oxide for high-performance supercapacitors. J. Mat. Chem. A.

[74] He X., Wang W.-N. (2019). Synthesis of Cu-Trimesic Acid/Cu-1,4-Benzenedioic Acid via Microdroplets: Role of Component Compositions. Cryst. Growth Des..

[75] Venu B., Shirisha V., Vishali B., Naresh G., Kishore R., Sreedhar I., Venugopal A. (2020). A Cu-BTC metal–organic framework (MOF) as an efficient heterogeneous catalyst for the aerobic oxidative synthesis of imines from primary amines under solvent free conditions. New J. Chem..

[76] Riou-Cavellec M., Albinet C., Greneche J.-M., Ferey G. (2001). Study of the iron/trimesic acid system for the hydrothermal synthesis of hybrid materials. J. Mat. Chem..

[77] Fang Y., Yang Z., Liu X. (2020). MIL-100(Fe) and its derivatives: From synthesis to application for wastewater decontamination. Environ. Sci. Pollut. Res..

[78] Addison A.W., Rao T.N., Reedijk J., van Rijn J., Verschoor G.C. (1984). Synthesis, structure, and spectroscopic properties of copper(II) compounds containing nitrogen–sulphur donor ligands; the crystal and molecular structure of aqua [1,7-bis(N-methylbenzimidazol-2′-yl)-2,6-dithiaheptane]copper(II) perchlorate. J. Chem. Soc. Dalton Trans..

[79] Alexandrov E.V., Blatov V.A., Kochetkov A.V., Proserpio D.M. (2011). Underlying nets in three-periodic coordination polymers: Topology, taxonomy and prediction from a computer-aided analysis of the Cambridge Structural Database. Cryst. Eng. Comm..

[80] Blatov V.A., Shevchenko A.P., Proserpio D.M. (2014). Applied topological analysis of crystal structures with the program package ToposPro. Cryst. Growth Des..

[81] O’Keeffe M., Peskov M.A., Ramsden S.J., Yaghi O.M. (2008). The reticular chemistry structure resource (RCSR) database of, and symbols for, crystal nets. Acc. Chem. Res..

[82] Ahamad M.N., Khan M.S., Shahid M., Ashmad M. (2020). Metal organic frameworks decorated with free carboxylic acid groups: Topology, metal capture and dye adsorption properties. Dalton Trans..

[83] Ahamad M.N., Shahid M., Ashmad M., Sama F. (2019). Cu(II) MOFs Based on Bipyridyls: Topology, Magnetism, and Exploring Sensing Ability toward Multiple Nitroaromatic Explosives. ACS Omega.

[84] Li J.-X., Qin Z.-B., Li Y.-H., Cui G.-H. (2018). Two luminescent Cd(II)-MOFs based on bis(benzimidazole) and aromatic dicarboxylate ligands as chemosensor for highly selective sensing of Fe^3+^. Polyhedron.

[85] Yang Q., Zhao J.-P., Liu Z.-Y. (2012). Single crystal to single crystal transition in (10, 3)-d framework with pyrazine-2-carboxylate ligand: Synthesis, structures and magnetism. J. Solid State Chem..

[86] Gabriel C., Vangelis A.A., Raptopoulou C.P., Terzis A., Psycharis V., Zervou M., Bertmer M., Salifoglou A. (2015). Structural–Spectrochemical Correlations of Variable Dimensionality Crystalline Metal–Organic Framework Materials in Hydrothermal Reactivity Patterns of Binary–Ternary Systems of Pb(II) with (a)Cyclic (Poly)carboxylate and Aromatic Chelator Ligands. Cryst. Growth Des..

[87] Wang L., Xue R., Li Y., Zhao Y., Liu F., Huang K. (2014). Hydrogen-bonding patterns in a series of multi-component molecular solids formed by 2,3,5,6-tetramethylpyrazine with selected carboxylic acids. Cryst. Eng. Comm..

[88] Chilton N.F., Anderson R.P., Turner L.D., Soncini A., Murray K.S. (2013). PHI: A powerful new program for the analysis of anisotropic monomeric and exchange--coupled polynuclear d-- and f--block complexes. J. Comput. Chem..

[89] Hafez R.S., El-Khiyami S. (2020). Effect of copper (II) nitrate 3H_2_O on the crystalline, optical and electrical properties of poly(vinyl alcohol) films. J. Polym. Res..

[90] Castro I., Faus J., Julve M., Amigo J., Sletten J., Debaerdemaeker T. (1990). Copper(II)-assisted hydrolysis of 2,4,6-tris(2-pyridyl)-1,3,5-triazine. Part 3. Crystal structures of diaqua[bis(2-pyridylcarbonyl)amido]copper(II) nitrate dihydrate and aquabis(pyridine-2-carboxamide)copper(II) nitrate monohydrate. Dalton Trans..

[91] Milios C.J., Raptopoulou C.P., Terzis A., Vicente R., Escuer A., Perlepes S.P. (2003). Di-2-pyridyl ketone oxime in 3d-metal carboxylate cluster chemistry: A new family of mixed-valence Mn_2_^II^Mn_2_^III^ complexes. Inorg. Chem. Commun..

[92] Bernasek E. (1957). Pyridineamidoximes. J. Org. Chem..

[93] Orama M., Saarinen H., Korvenranta J. (1990). Formation of trinuclear copper(II) complexes with three pyridine oxime ligands in aqueous solution. J. Coord. Chem..

[94] Sheldrick G.M. (2015). SHELXT—Integrated space-group and crystal-structure determination. Acta Cryst..

[95] McArdle P., Gilligan K., Cunningham D., Dark R., Mahon M. (2004). A method for the prediction of the crystal structure of ionic organic compounds—The crystal structures of o-toluidinium chloride and bromide and polymorphism of bicifadine hydrochloride. Cryst. Eng. Comm..

[96] Brandenburg K. (2006). DIAMOND.

