# Supplementary material for: ZnII and CuII-Based Coordination Polymers and Metal Organic Frameworks by the of Use of 2-Pyridyl Oximes and 1,3,5-Benzenetricarboxylic Acid
Source: Molecules. 2021 Jan 18;26(2):491. doi: 10.3390/molecules26020491 (PMC7831896; doi:10.3390/molecules26020491)
Supplement: Supplementary file 1 [file molecules-26-00491-s001.pdf]

Supplementary Materials

Zn^{II} and Cu^{II}-Based Coordination Polymers and Metal Organic Frameworks by the of Use of 2-Pyridyl Oximes and 1,3,5-Benzenetricarboxylic Acid

Ioannis Mylonas-Margaritis [1], Julia Mayans [2], Patrick McArdle [1] and Constantina Papatriantafyllopoulou [1,*]

[1] School of Chemistry, College of Science and Engineering, National University of Ireland Galway, University Road, H91 TK33 Galway, Ireland; i.mylonas-margaritis1@nuigalway.ie (I.M.-M.); patrick.mcardle@nuigalway.ie (P.M.A)
[2] Instituto de Ciencia Molecular (ICMol), Universidad de Valencia, Catedrático José Beltran 2, 46980 Paterna Valencia, Spain; julia.mayans@qi.ub.edu
* Correspondence: constantina.papatriantafyllopo@nuigalway.ie; Tel.: +353-91-493-462

Table S1. Selected interatomic distances (Å) and angles for **1**·$2H_2O$.

**Bonds**			
Zn1-N5	2.110(8)	Zn1-N2	2.134(7)
Zn1-N4	2.151(5)	Zn1-O3	2.118(4)
Zn1-O9	2.102(4)	Zn1-N1	2.144(6)
**Angles**			
N5-Zn1-N4	76.0(2)	N1-Zn1-O3	87.4(2)
N4-Zn1-N2	97.2(2)	N1-Zn1-N5	99.6(2)
N2-Zn1-O3	88.7(2)	O4-Zn1-N4	86.2(2)
O3-Zn1-N5	98.4(2)	O4-Zn1-N2	96.7(2)
N1-Zn1-N4	96.3(2)	O4-Zn1-N5	88.7(2)
N1-Zn1-N2	75.1(2)	O4-Zn1-O3	90.9(2)

Table S2. Selected interatomic distances (Å) and angles for **2**.

**Bonds**			
Zn1-N6	2.091(3)	Zn1-O3	2.055(3)
Zn1-N4	2.282(3)	Zn1-N3	2.111(3)
Zn1-N1	2.172(3)	Zn1-O6	2.084(2)
**Angles**			
N4-Zn1-N1	89.7(1)	N6-Zn1-O6	104.0(1)
N1-Zn1-O6	92.2(1)	N6-Zn1-O3	94.1(1)
O6-Zn1-O3	88.6(1)	N3-Zn1-N4	87.3(1)
O3-Zn1-N4	90.1(1)	N3-Zn1-O3	92.8(1)
N6-Zn1-N1	97.1(1)	N3-Zn1-O6	95.6(1)
N6-Zn1-N4	73.3(1)	N3-Zn1-N1	75.0(1)

Table S3. Selected interatomic distances (Å) and angles for **3**.

**Bonds**			
Cu1-N1	2.006(2)	Cu1-N3	1.977(3)
Cu1-O5	1.942(2)	Cu1-O1	1.918(2)
Cu1-O7	2.629(2)		
**Angles**			
N3-Cu1-O5	170.69	N1-Cu1-N3	79.61
O5-Cu1-O7	97.62	O1-Cu1-O5	87.79
O7-Cu1-N3	83.43	O1-Cu1-O7	88.51
N1-Cu1-O5	91.09	O1-Cu1-N3	101.5
N1-Cu1-O7	97.68		

Table S4. Selected interatomic distances (Å) and angles for **4**.

**Bonds**			
Cu1-O2	1.924(5)	Cu1-O5	1.934(4)
Cu1-N2	1.982(4)	Cu1-N1	2.001(6)
**Angles**			
O2-Cu1-N2	101.8(2)	N1-Cu1-O5	90.8(2)
N2-Cu1-N1	79.8(2)	O5-Cu1-O2	88.6(2)

Table S5. Selected interatomic distances (Å) and angles for **5**$\cdot 4H_2O$.

**Bonds**			
Cu1-N2	2.025(2)	Cu1-O2	2.260(2)
Cu1-N1	2.004(3)	Cu1-O6	1.951(2)
Cu1-O3	1.948(2)		
**Angles**			
O3-Cu1-N1	174.64(8	N2-Cu1-O3	101.57(9)
N1-Cu1-O2	89.09(8)	O6-Cu1-N1	91.78(8)
O2-Cu1-O3	85.60(8)	O6-Cu1-O2	99.72(8)
N2-Cu1-N1	78.72(9)	O6-Cu1-O3	89.88(8)
N2-Cu1-O2	101.49(9)	N2-Cu1-O6	156.55(9)
N1-Cu1-O3	174.64(8)		

Table S6. Hydrogen bonding details for **2**[a].

D-H···A	D···A (Å)	H···A (Å)	DHA (°)	Symmetry operator of A
O1-H1O1···O4	2.648	1.789	165.91	x, y, z
N5-H1N5···O4	2.894	2.147	163.53	1-x, -1/2+y, 1.5-z
O5-H1O5···O8	2.655	1.876	145.31	-x, -1/2+y, 1/2-z

[a] A=acceptor, D=donor.

Table S7. Intermolecular hydrogen bonding details for **5**·$4H_2O$ [a].

D-H···A	D···A (Å)	H···A (Å)	DHA (°)	Symmetry operator of A
O2-H2O2···O9	2.743	1.907	170.95	x, y, z
O8-H1O8···O10	2.560	1.69	174.02	x, y, z
O1-H1O1···O4	2.510	1.616	174.08	x, y, z
O2-H2O2···O7	2.776	2.061	169.55	x, y, -1+z

[a] A=acceptor, D=donor.

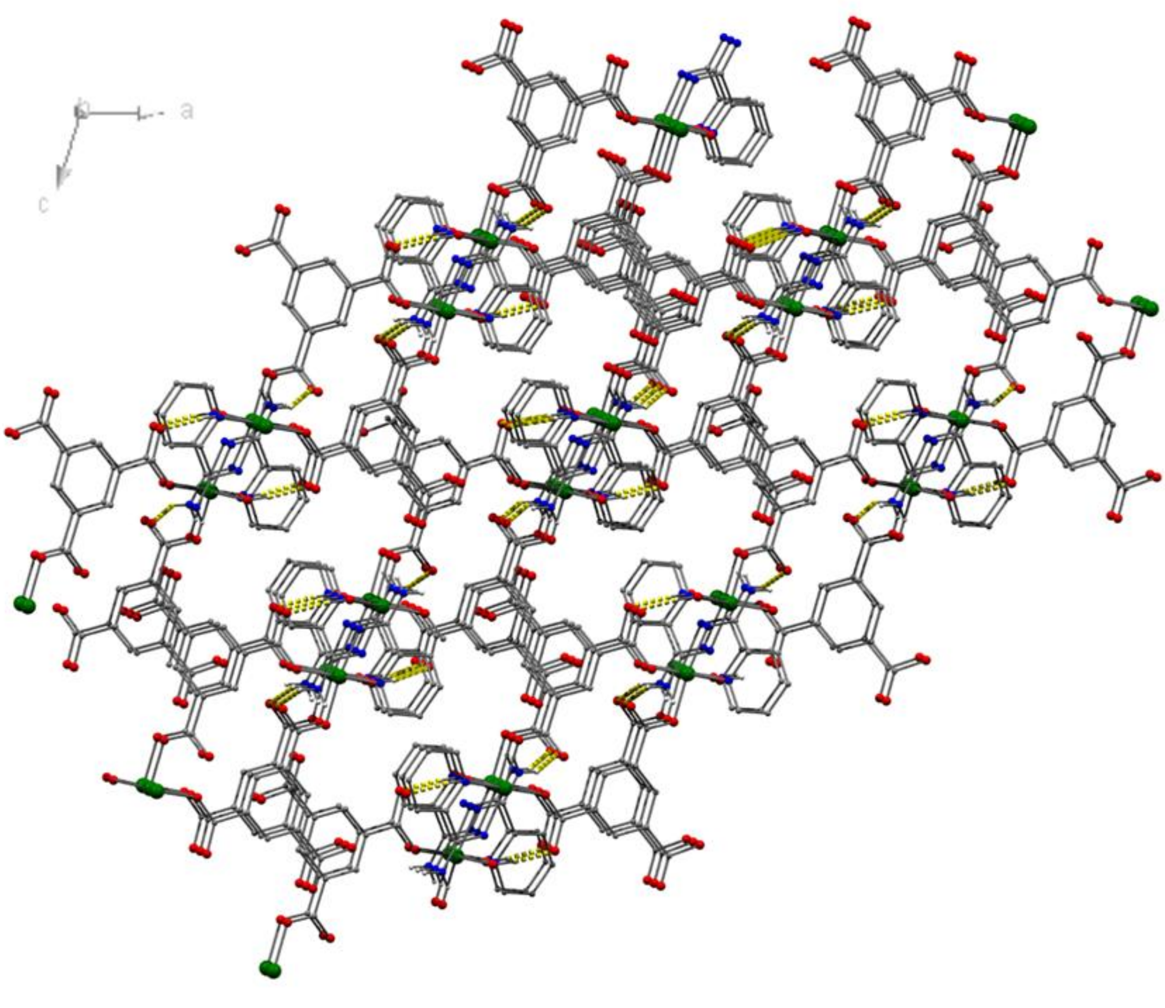

Figure S1. Representation of the 3D network formed through hydrogen bonding interactions in **3**.

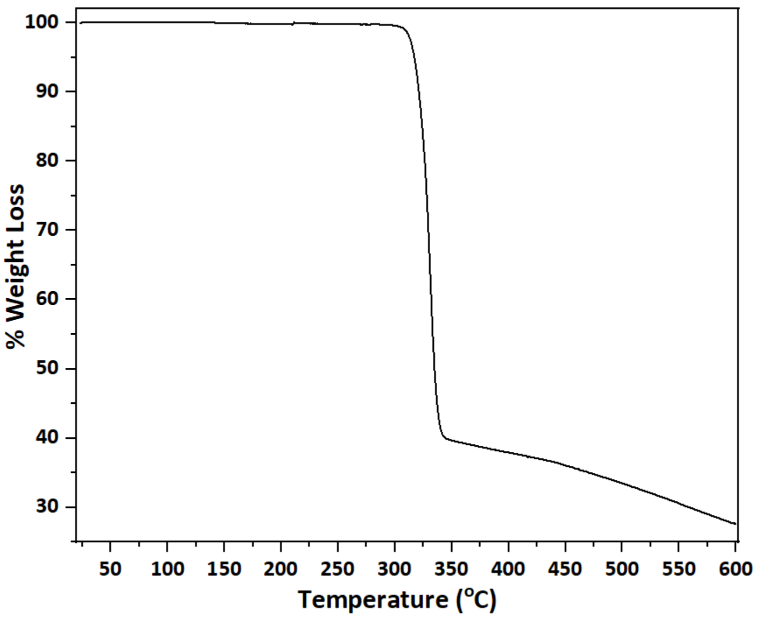

Figure S2. The TGA plot for **3**.

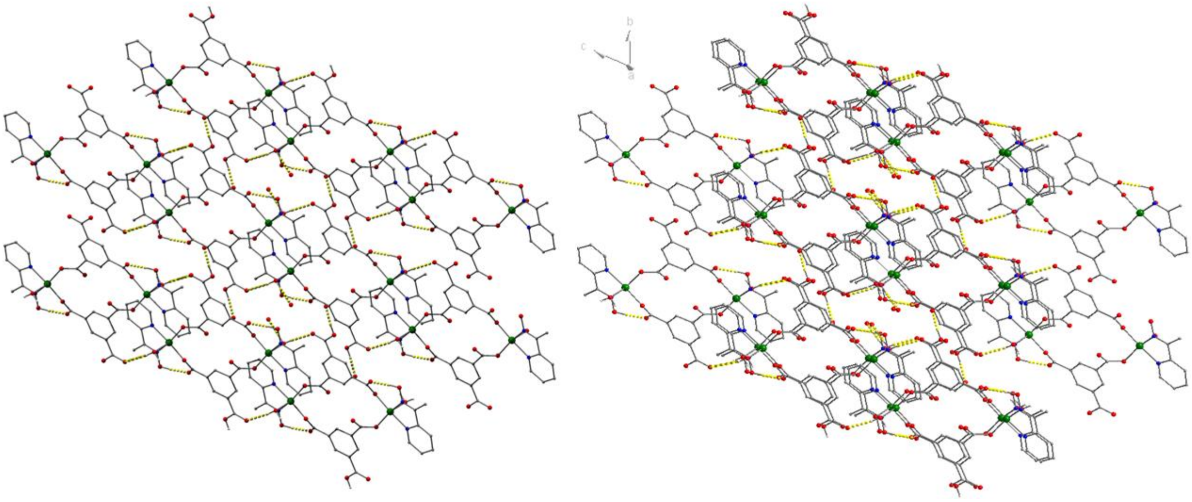

Figure S3. Representation of the 3D network formed through hydrogen bonding interactions in **5**·$4H_2O$.

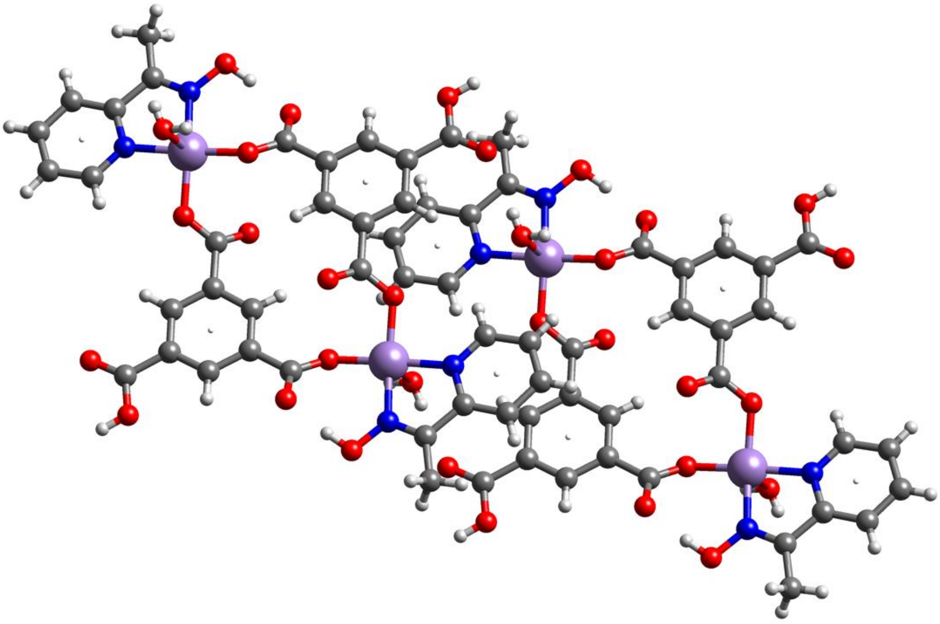

Figure S4. Representation of the intermolecular π-π stacking interactions in **5**·$4H_2O$. The centroids of the interacting aromatic rings are represented with white spheres.

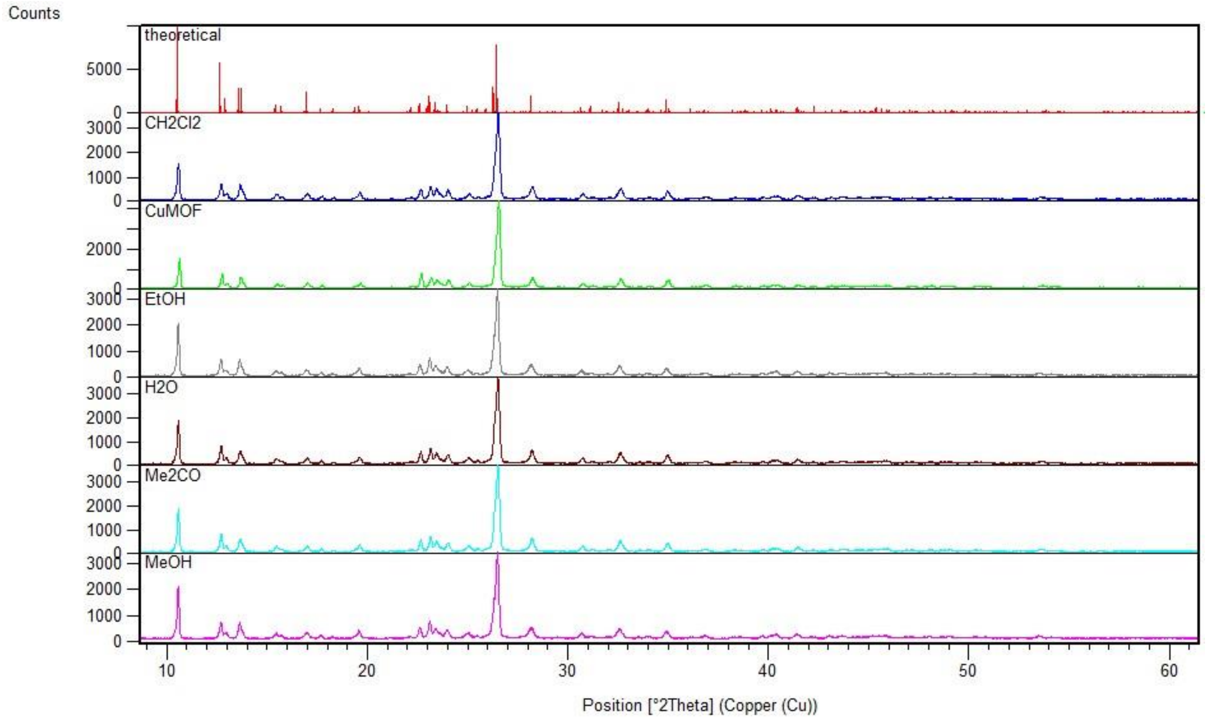

Figure S5. Comparison of the pxrd patterns for **3** (theoretical, red; experimental: green; in CH_2Cl_2, navy blue; in EtOH, grey; in H_2O, magenta; in Me_2CO, cyan; in MeOH, pink.

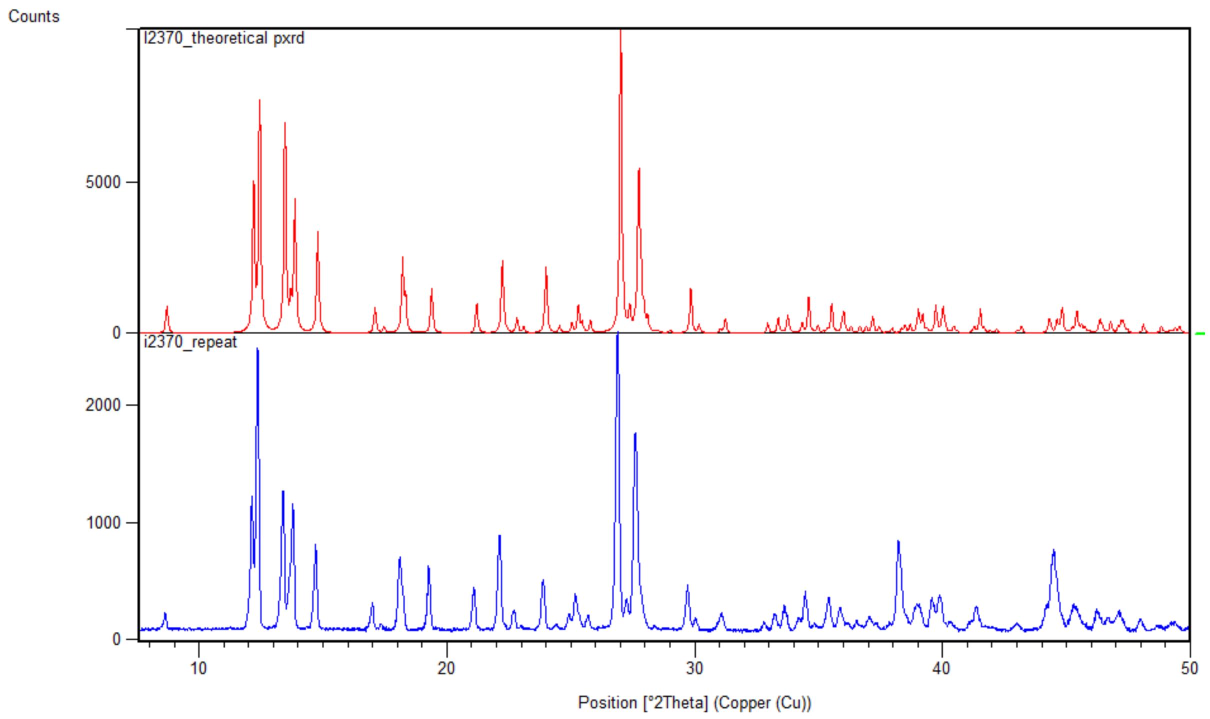

Figure S6. Comparison of the pxrd patterns for **5**·$4H_2O$ (theoretical, red; experimental, blue).

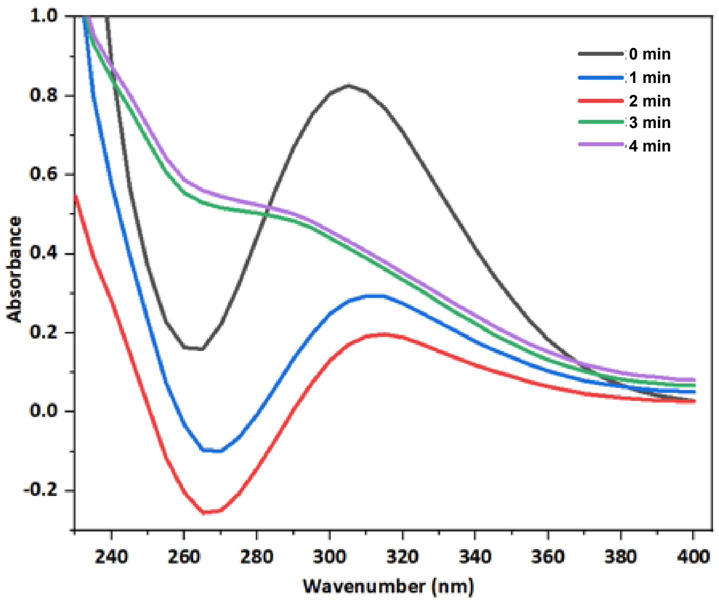

Figure S7. UV-vis plot for the adsorption of iron(III) nitrate nonahydrate by **3** in H_2O. Initial concentration: 0.10 mmol $Fe(NO_3)_3$ in 10 ml of H_2O.

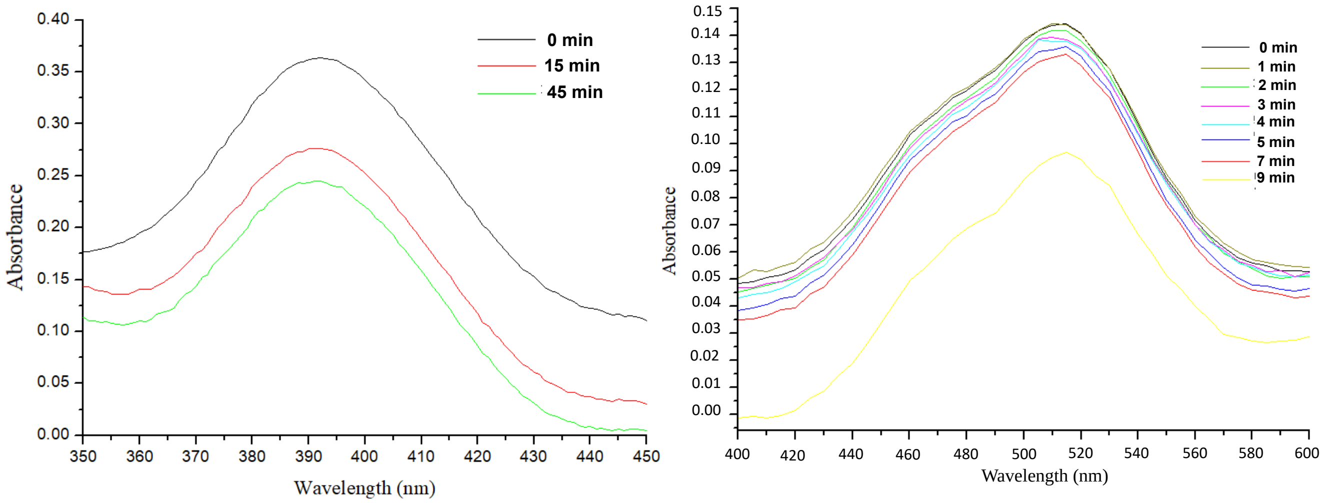

Figure S8. Preliminary results on the adsorption of $Ni(NO_3)_2$ (left) and $Co(NO_3)_2$ (right) by **3** in H_2O.

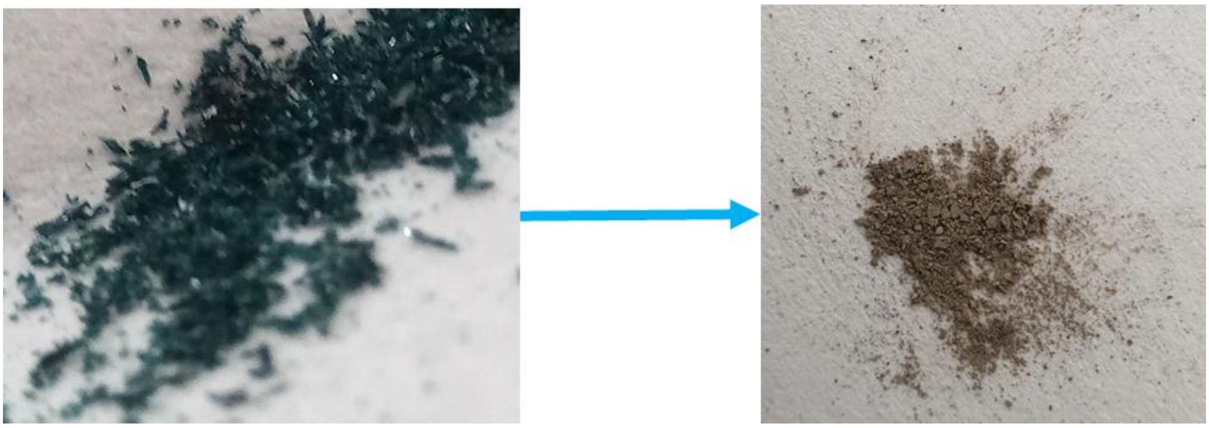

Figure S9. Photo of crystals of **3** (left) before the reaction and the formed brown compound (right).

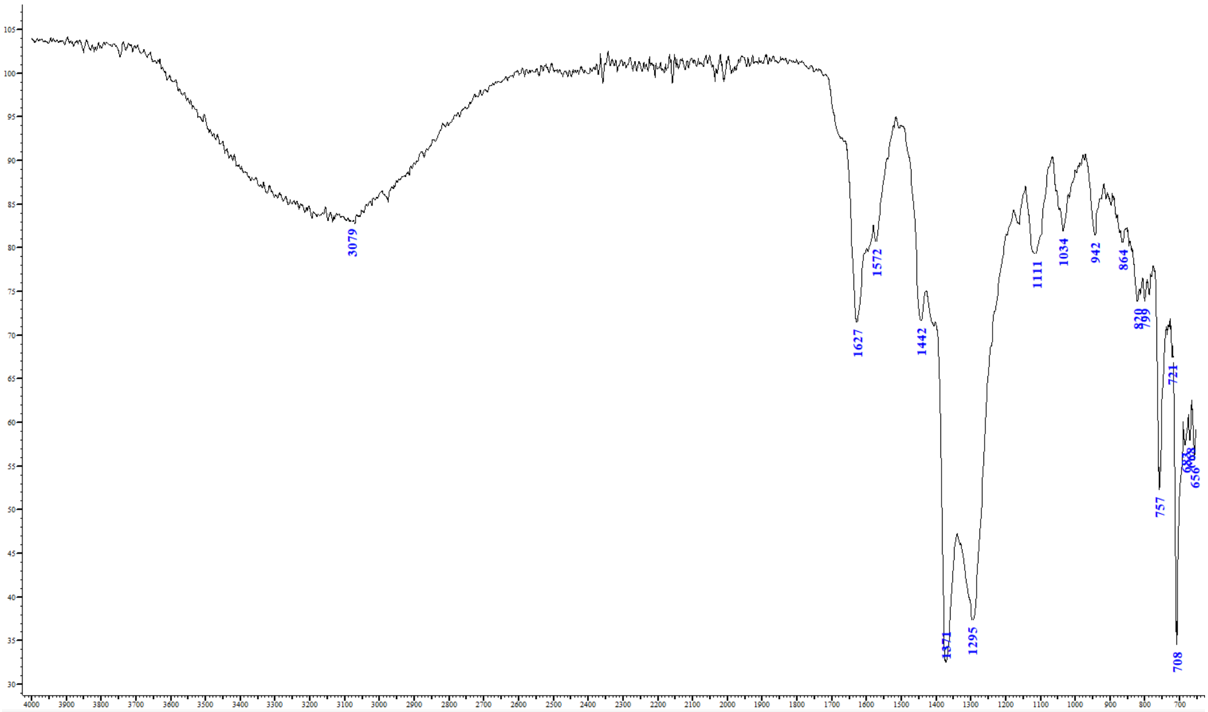

Figure S10. The infrared spectra of the isolated brown precipitated.
